# Modeling the impact of digital readiness in recruiting and sustaining underrepresented groups: Data from the *All of Us* research program

**DOI:** 10.3389/fdgth.2022.1082098

**Published:** 2023-04-13

**Authors:** Soumya Kini, Dave Duluk, Joshua Weinstein

**Affiliations:** ^1^ Principal Health Systems Engineer at The MITRE Corporation, Mclean, VA, United States; ^2^ Lead Data Scientist at The MITRE Corporation, Mclean, VA, United States; ^3^ Department of Health Policy and Management, Gillings School of Global Public Health, University of North Carolina at Chapel Hill, Chapel Hill, NC, United States

**Keywords:** longitudinal data collection, underrepresented in biomedical research (UBR), national institutes of health (NIH), diversity, *All of Us* research program, healthcare, health disparities, health equity, digital readiness

## Abstract

The All of Us Research Program (*All of Us* or Program) is an ongoing longitudinal data collection operated by the National Institutes of Health (NIH). The Program aims to improve healthcare for all through the development of a biomedical research resource reflective of the diversity of the United States that includes Underrepresented in Biomedical Research (UBR) groups. Federally Qualified Health Centers (FQHCs) are a key recruitment stream of UBR participants, which are community based and provide primary care and preventive services in medically underserved areas. Over 90% of FQHC patients enrolled in *All of Us* to date are UBR. The COVID-19 pandemic caused a pause in *All of Us* activities. Re-starting the activities was a challenge, especially due to the digital divide faced by FQHC participants, and that most Program activities are primarily completed *via* web-based portal from a computer or a mobile device. This paper investigates the extent to which digital readiness impacted recruitment and sustainment of a pre-pandemic sample of 2,791 FQHC participants to the Program. Digital readiness was defined by access to home-based or other internet-accessing devices, and participants’ comfort level using such devices. Results from multivariable logistic regression models showed that lower age, more education, female gender identity, and higher income were associated with higher digital readiness (*p* ≤ 0.01). Race, rurality, and sexual orientation status were not significant factors associated with digital readiness. Older participants had higher odds of completing Program activities, even though less digitally ready than their younger peers, as they often completed the activities during their in-person clinical visits. A subsequent weighted model demonstrated that FQHC participants who were digitally ready had 27% higher odds of completing Program activities than those not digitally ready. The data highlight the need for improved connectivity and sustainment between longitudinal data collection, research programs, and UBR participants, particularly among those facing the digital divide. Quantifying digital challenges provide operational insights for longitudinal data collection (*All of Us*, or others), and broadly, other aspects of digital medicine such as telehealth or patient portals by recognizing digital readiness of participants and patients, and the level of support required for success.

## 1. Introduction

The *All of Us* Research Program (*All of Us* or Program) is an ongoing longitudinal data collection operated by the National Institutes of Health (NIH) to collect lifestyle, health, socioeconomic, environmental, and biological data from 1 million United States-based participants (1). Diversity is a core tenet of the Program, which aims to ensure those who are typically Underrepresented in Biomedical Research (UBR) are the majority of those enrolled and retained (2).

The Program has defined specific UBR categories that include racial identity, age when consented to Program participation, biological sex at birth, sexual orientation, gender identity, income, educational attainment, access to care, disability and rurality. Three site types are responsible for enrolling and retaining these participants: Regional Medical Centers, Veterans Administration Medical Centers, and Federally Qualified Health Centers (FQHCs). FQHCs are a key recruitment stream of UBR participants and are centrally coordinated and supported by The MITRE Corporation (MITRE) (3). FQHCs are community based and provide primary care and preventive services in medically underserved areas regardless of ability to pay (4). Over 90% of the FQHC patients are low income, over 80% are publicly insured or uninsured, and the majority are members of racial and ethnic minority groups (5). Individuals and families served by FQHCs are among the most economically vulnerable in the nation and often have complex health and social challenges. Enrollment activities for the Program are primarily completed *via* a web-based portal from a computer or a mobile device. Therefore, digital readiness plays a key role in the FQHC *All of Us* team's ability to enroll and retain participants in the Program. Barriers for utilizing digital devices among patients at FQHCs include cost and lack of information, access to technology, and broadband connection. Digital health device adoption at FQHCs requires education, investment, and high-touch methods (6).

While the Program intends to ensure enrichment of UBR populations, historically the recruitment of UBR populations (particularly racial and ethnic minorities, and low-income communities) to clinical studies is largely viewed as a challenge (7). For example, where race and ethnicity are concerned, though African Americans and Hispanics represent 13% and 16% of the United States population, respectively, only 5% of clinical trial participants are African American and 1% of participants are Hispanic (8). Socially and economically disadvantaged groups are least likely to have access to a smartphone, computer, home broadband, or internet. For example, a 2021 study published by the Pew Research Center found that 13% of low-income adults do not have access to a smartphone, computer, or home broadband, in comparison to 1% of those with incomes over $100,000 (9). Populations that discontinue internet use due to cost and disability are more likely to be Hispanic, Black, or low income. Telemedicine is also less adopted by UBR groups, including those who are older, are racial and ethnic minorities, have a rural residence, and are publicly insured (10). A Brookings Institute report published in 2020 found that, when examining the issue by income groups, 38% of households earning less than $20,000 lack a broadband subscription (11). This is a typical manifestation of the digital divide, defined as the gap between those who have and those who do not have access to information technology. Digital exclusion can limit participation in clinical research studies, innovative clinical trial design, and the collection of patient-reported outcomes. Furthermore, while digital exclusion is concentrated among the poorest, least educated, disabled, and socially isolated, these groups also gain less benefit from the use of digital technology in their health outcomes than do their more privileged peers. In this manner, digital exclusion compounds poor health outcomes, and is now termed a new social determinant of health (12–16).

This paper investigates the extent to which digital readiness impacts recruitment and sustainment of participants to the Program who are patients at FQHCs, particularly among UBR groups. Analyses contained in this paper provide operational insights for NIH, healthcare providers, and researchers on developing and adopting a digital inclusion-informed strategy that recognizes the digital readiness of participants and patients, and the level of staff support required for a broad range of activities, such as recruiting for longitudinal data collection and studies, telehealth, telemedicine, or patient portals.

## 2. Methods

The analyses utilize quantitative data on adult FQHC patients who are *All of Us* participants; data are housed at the Data and Research Center (DRC) Program Data Repository. The DRC is located at Vanderbilt University Medical Center and is funded by the NIH Program (17). Quantitative data used in this paper include participant demographics, *All of Us* operational data, and a participant survey questionnaire called the Minimum Common Metrics (MCM) collected by the following six FQHCs from across the country that reflect the diversity of the United States: Community Health Center, Inc. located in Connecticut; Cherokee Health Systems located in Tennessee; Cooperative Health located in South Carolina; Jackson-Hinds Comprehensive Health Center located in Mississippi; Sun River Health located in New York, and San Ysidro Health located in California. Two additional FQHCs located in Hawaii and Puerto Rico have since been added in 2021 and 2022, respectively. These two FQHCs had not begun collecting MCM data at the time of writing this paper and are therefore excluded from the analysis.

On March 16, 2020, NIH paused in-person Program activities to assist in preventing the spread of COVID-19 (23). UBR participants were disproportionality impacted during the COVID-19 pause of in-person activities. FQHCs adopted virtual strategies using Computer Assisted Telephone Interviewing (CATI), which was launched by *All of Us* in January 2021. CATI has presented a new opportunity for FQHCs to contact participants *via* phone and record participant responses to surveys in real time. Follow-up research is underway to characterize the *All of Us* participants who utilized CATI at FQHCs as well as to explore the relationship between retention activities *via* CATI and UBR status. The quantitative data collection methods were performed in accordance with relevant guidelines and regulations and approved by All of Us Research Program Institutional Review Board (IRB00010472). The participants included in this paper have provided consent to having their data used for research. All data used were derived from participants who provided written consent on or before April 28, 2022, which is the freeze date for the dataset used in this study. Variables in the dataset are described in the sections below.

### 2.1. Participant demographics and recruitment data

Participant demographics include data that the Program considers for determining UBR and are collected from FQHC patients at the time of registration. They include racial identity, age when consented to Program participation, biological sex at birth, sexual orientation, gender identity, income, educational attainment, and rurality at the time of writing this paper. Participants are considered UBR if one or more of the definitions provided in Table 1 is true.

**Table 1:** *All of Us* definitions for participants that are Underrepresented in Biomedical Research (UBR).

UBR Category	Program Definition for UBR
Racial identity	Participant has identified as other than White. Also includes participants who self-identify as Hispanic, Latino, or Spanish
Age at consent	Participant is 65 years or older when they consented to Program participation
Sex at birth	Participant self-reports intersex as their biological sex at birth
Sexual orientation	Participant selects any sexual orientation choice other than straight
Gender identity	Participant selects any gender identity choice other than man or woman
Income	Participant's annual household income is less than $25,000 a year
Educational attainment	Participant does not have a high school diploma or General Educational Development (GED)
Rurality	Participant is a resident of an established rural and non-metropolitan ZIP code, based on the Health Resources and Services Administration Federal Office of Rural Health Policy data files

Recruitment data include participants who are considered retained per the Program definition at the time of writing this paper, by completing the activities outlined in Table 2. In *All of Us*, retained participants complete follow-up surveys at least once every 18 months after their enrollment. In this context, retention provides a measure for the ability of the FQHCs to sustain engagement with participants after recruitment to the Program. All activities, except submitting bio samples to the Biobank, are completed by participants on a web-based portal when they come in-person to the FQHCs or virtually from a computer or a mobile device.

**Table 2:** Required Actions to be Completed by the Participant in the *All of Us* Research Program.

Action	Activity Type
Create an account (i.e., *has a participant ID*)	Enrollment
Consent to program participation	
Consent to EHR data sharing	
Complete the Basics Survey	
Complete the Overall Health Survey	
Complete the Lifestyle Survey	
Have Biobank receipt of sample (blood, urine or saliva)	
Complete Physical measurements	
Complete the Social Determinants of Health Survey	Retention
Complete the Health Care Access Survey	
Complete the Family Health Survey	
Complete the Medical History Survey	
Complete the COVID-19 Participant Experience Survey (*retired in 2021*)	
Consent to return of genetic results	
Update consent for Program participation	
Complete the Minute Survey on COVID-19 Vaccines (*retired in 2022*)	

### 2.2. Minimum common metrics data

MCM is an Institutional Review Board (IRB)-approved questionnaire collected by FQHCs for MITRE. It contains participant responses on their enrollment experience, digital readiness, access to a fitness tracker, and level of FQHC staff assistance required for completing *All of Us* activities. Answers to these questions are collected throughout the participant journey (Table 2), with a goal of understanding FQHC participant experiences and resources available for them to participate in the Program. Table 3 provides all questions asked of participants in the MCM data at the time of writing this paper. The MCM survey questions were asked by FQHC staff to all participants at their time of enrollment in *All of Us*. However, per the IRB requirements, participants were given a choice to decline responding to the MCM survey entirely or skip any of the questions. A subset of responses to the MCM questions that pertain to participants' digital readiness were used in the analysis contained in this paper.

**Table 3:** FQHC MCM questionnaire.

Category	What Is Asked
Enrollment Experience	• How did you first hear about the Program? • What would you say is your main reason for wanting to join the Program? • Research assistant created email account to enroll (*filled out by FQHC staff member*)
Digital Readiness	• Do you have access to a computer, tablet, or mobile phone at home? • Do you have access to the internet through Wi-Fi or mobile data at home? • How comfortable are you using technology, such as navigating emails, answering survey questions, or navigating a patient account portal?
Fitness Tracker Access	• Do you have a fitness tracker (such as a FitBit, an Apple Watch, an app on your phone, etc.)? • [If yes] Have you linked/connected your fitness tracker to the *All of Us* Research Program Portal?
Level of FQHC Staff Support *(filled out by FQHC staff member; answer choices: assisted, facilitated, independent on-site, independent off-site, assisted virtual, facilitated virtual)*	• Level of FQHC staff support required to complete consent form for Program participation • Level of FQHC staff support required to complete consent form for EHR data sharing • Level of FQHC staff support required to complete consent form for return of genetic results • Level of FQHC staff support required to complete various required participant surveys

### 2.3. Qualitative data from FQHCs

In addition to quantitative data described in the previous sections, the MITRE team engaged the FQHC staff to collect qualitative data about their experiences and strategies in engaging population groups for retention activities. The MITRE team held a focus group with the FQHC staff in June 2022 to gather inputs. *All of Us* staff members from all six FQHCs participated in the focus group. Focus group discussion included open-ended conversations on the following topics: strategies FQHCs used to retain participants with low digital readiness into *All of Us*, and data sources FQHCs utilized to be better informed and to develop strategies for engaging population groups with low digital readiness. The discussions were focused on pre-pandemic scenario, given the scope of this paper. The MITRE team recorded the conversation upon consent from the FQHC staff members participating in the meeting and transcribed notes summarizing the conversations. Information collected from these questions were analyzed alongside quantitative results to develop insights on whether specific population groups that the model found significant for indicating low digital readiness influenced the FQHC engagement strategy.

### 2.4. Study population

The study population included 2,897 *All of Us* participants who responded to questions on digital readiness (three questions, Table 3) for the time-period between June 2019 and March 2020 when they completed required actions to become an enrolled participant (Table 2). This time-period was selected based on when the digital readiness questions were first asked by FQHCs (June 2019) to newly enrolled participants until the start of the COVID-19 pandemic (March 2020). The COVID-19 pandemic significantly changed the operational workflow at the FQHCs when NIH paused in-person *All of Us* activities. Therefore, data collected during the COVID-19 pandemic were not included in this analysis. Demographic variables, retention data, and participant responses to the MCM technology access questions were additional fields associated with the participants in the study sample.

### 2.5. Analytical methods

For purposes of this study, digital readiness was defined by access to home-based or other internet-accessing devices (computers, tablets, mobile phones, and other devices) and participants' comfort level using such devices. Responses to the three MCM technology access questions shown in Table 4 were utilized to define digital readiness. Participants who skipped or selected the “Prefer not to answer” option to any of the three questions were excluded from the analytic sample within the study time-period range since it was not possible to infer the digital readiness disposition for these participants. This reduced the final analytic sample from 2,897 to 2,791 participants. FQHC participant demographic distributions of those included in the analytic sample were compared with those who were excluded from the study sample to verify that the analytic sample was not a biased set relative to the larger FQHC *All of Us* population. All analyses in this paper were conducted using R and RStudio (18, 19). Any group with less than 20 participants were included in “Other” group to stay consistent with the Program data suppression levels to support data privacy.

**Table 4:** Answer choices for the MCM technology access questions.

Question #	Question Wording	Answer Choices
1	Do you have access to a computer, tablet, or mobile phone at home?	⬜ Yes ⬜ Intermittent ⬜ No ⬜ Prefer not to answer
2	Do you have access to the internet through Wi-Fi or mobile data at home?	⬜ Yes ⬜ Intermittent ⬜ No ⬜ Prefer not to answer
3	How comfortable are you using technology, such as navigating emails, answering survey questions, or navigating a patient account portal?	⬜ Very comfortable ⬜ Somewhat comfortable ⬜ Neutral ⬜ Somewhat uncomfortable ⬜ Not at all comfortable ⬜ Prefer not to answer

Participants who responded with a “Yes” or “Intermittent” to Questions 1 and 2, and “Very comfortable,” “Somewhat comfortable,” or “Neutral” to Question 3 were considered as digitally ready and transformed as such in the analytical data set. Participants who responded with a “No” to Questions 1 or 2 or “Somewhat uncomfortable” or “Not at all comfortable” to Question 3 were considered as not digitally ready and transformed as such in the analytical data set.

Retention data was transformed into a Boolean (1 or 0) variable, which was equal to 1 if the participant had ever been retained by completing the required activities outlined in Table 2 at some point during their involvement in the Program, and a 0 if the Participant was never retained because they did not complete any of the required activities since becoming an enrolled Participant during the study period.

#### 2.5.1. Characteristics of digitally ready groups in the program at FQHCs

The characteristics of the *All of Us* groups that are digitally ready (vs. not digitally ready) at FQHCs were explored using a multivariable logistic regression model (Model 1) with UBR variables of racial identity, age at consent, sex at birth, sexual orientation, gender identity, income, educational attainment, and rurality.

#### 2.5.2. Digital readiness impact on the retention of participants in the program at FQHCs

The effect of digital readiness on retention was measured using a second multivariable logistic regression model (Model 2). Inverse probability of treatment weights (IPTW) propensity score methods were used to create a weighted synthetic population. IPTW utilizes propensity scores to balance baseline characteristics in exposed and unexposed groups. Applied to the current study, IPTW balances UBR characteristics in the digitally ready and not digitally ready groups, therefore minimizing the impact of confounding due to those measured UBR characteristics (20). With this approach, one cannot attribute differences in retention outcomes due to differences in UBR characteristics between individuals who are digitally ready and those who are not digitally ready.

UBR variables significant at the 0.05 level from Model 1 were used in the propensity score model. Multivariable logistic regression was used to model participants' probability of retention as weighted by the previously described IPTW. Propensity score modeling was conducted using the WeightIt and Survey R packages (21, 22). Results from both weighted and unweighted models are included in the results.

## 3. Results and discussion

### 3.1. Digital readiness landscape of the program participants at FQHCs

**Figure 1:**
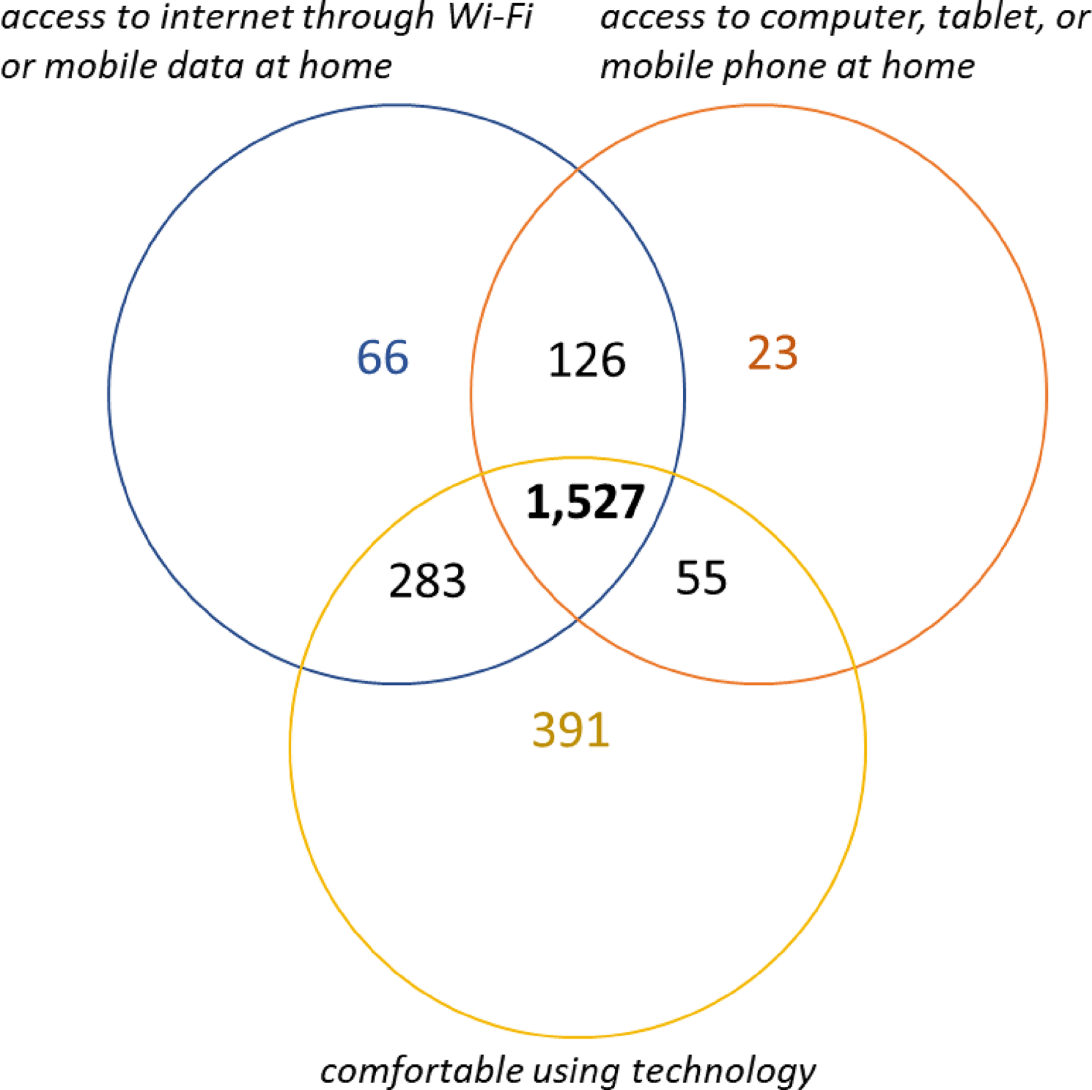
Digital readiness disposition among the *All of Us* participants at FQHCs. [Total number of participants = 2,791. Digital readiness was defined by access to home-based or other internet-accessing devices (computers, tablets, mobile phones, and other devices) and participants’ comfort level using such devices].

The analytic sample included 2,791 *All of Us* participants who responded to the three MCM digital readiness questions between June 2019 and March 2020 when they completed the required actions to become an enrolled Participant. Applying the definition of digital readiness described earlier in this paper to this sample resulted in 1,527 participants who were considered digitally ready and 1,264 participants who were considered not digitally ready, as shown in Figure 1.

Figure 1 indicates that, among the 1,264 Program participants who were not digitally ready, about 31% (391) were comfortable using technology but did not have access to the internet or a computer at home; and 22% (283) of participants were comfortable using technology and had access to the internet but did not have a computer, tablet, or mobile phone at home. These observations indicate that a majority of the participants (53%) were not digitally ready due to lack of a device, which they may not have been able to afford considering that over 90% of FQHC patients are low income (5).

Prior to setting up the model, FQHC participant demographic distributions of those included in the analytic sample were compared with those who were excluded to verify that the analytic sample was not a biased set relative to the larger FQHC *All of Us* population. The two groups were very similar in distribution (*χ*^2^ test *p* ≥ 0.13 for all comparison groups), indicating that specific demographic groups were not over- or under-represented in the analytic sample. Examination of demographic characteristics, to the extent they might result in collinearity, showed that gender identity and sex at birth were strongly associated (Cramer's *V* = 0.65). Therefore, gender identity was used in the final model as it represented the participant's self-identification; sex at birth was excluded.

**Figure 2:**
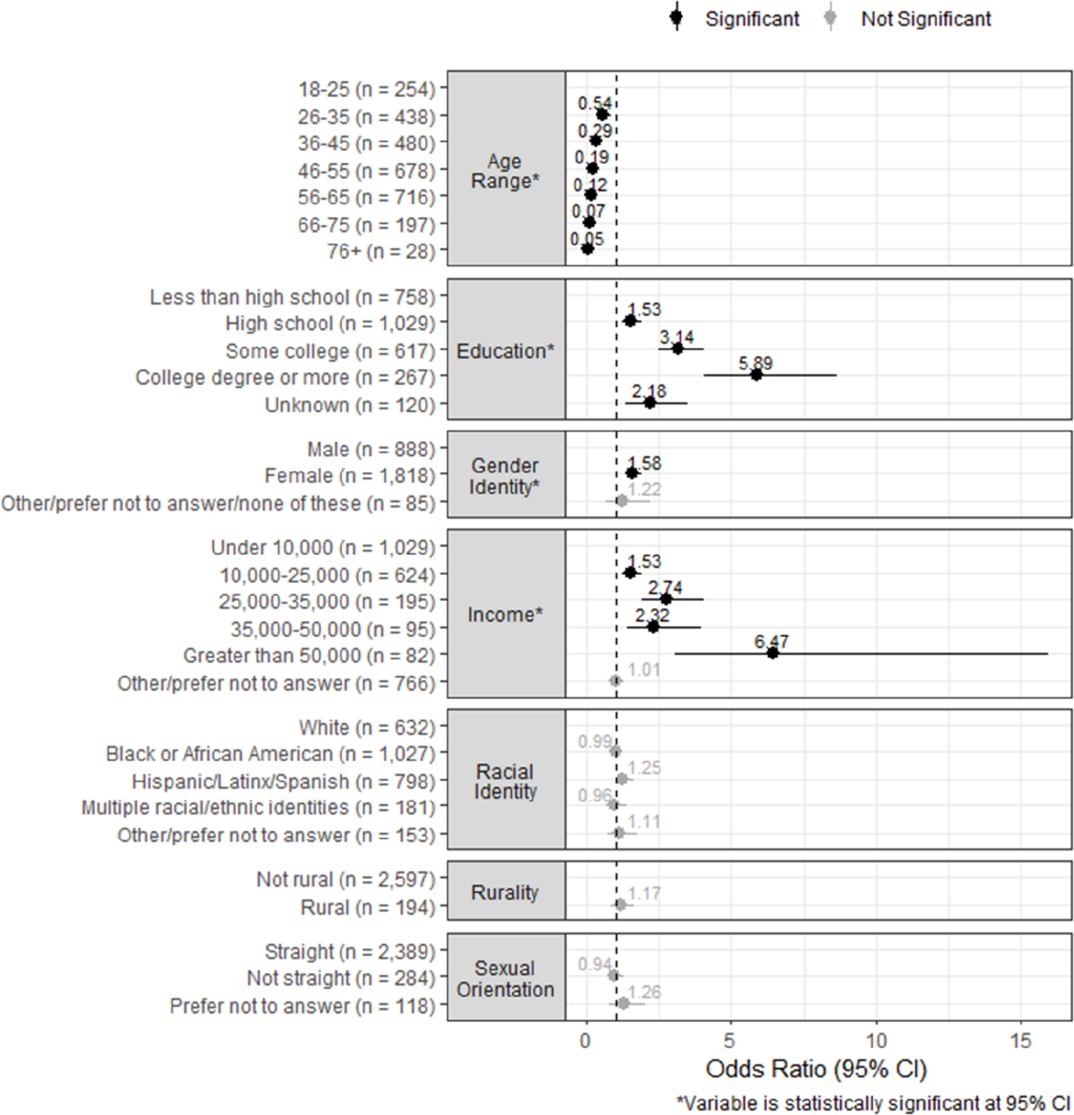
Results from the model to understand characteristics of groups that are not digitally ready at FQHCs. (Outcome variable: Digital Readiness. Coded as 1 if the participant was digitally ready, and 0 if the participant was not digitally ready. Digital readiness was defined by access to home-based or other internet-accessing devices (computers, tablets, mobile phones, and other devices) and participants' comfort level using such devices).

Results from a multivariable logistic regression model relating to characteristics of groups that are not digitally ready at FQHCs are shown in Figure 2 (Model 1). A complete table containing Odds Ratios (OR) and 95% Confidence Interval (CI) values is included in the Supplementary Table S1 of this paper.

Age at consent, gender identity, income, and educational attainment were the significant variables associated with digital readiness (*p *≤ 0.01). Race, rurality, and sexual orientation status were not significant factors associated with digital readiness. Participants who were 26–35 years of age when completing their primary consent had 46% lower odds of being digitally ready (OR 0.54) compared with their 18- to 25-year-old peers. This trend continued with every decade of age at consent increase. For example, participants who were 36–45 years of age at consent had 71% lower odds (OR 0.29) and those 46–55 years of age at consent had 81% lower odds (OR 0.19), peaking at 76 years or older of age at consent, who had 95% lower odds (OR 0.05) to be digitally ready.

Participants who identified as females had 60% higher odds of being digitally ready (OR 1.58) than those that identified as males. Further analyses showed that FQHC *All of Us* participants who identified as females were more digitally ready than males at all age groups, races, rurality, incomes, and education levels (data not shown).

Higher income levels were associated with higher digital readiness; participants with income levels greater than $50,000 had 6.5 times higher odds of being digitally ready (OR 6.47) than those with incomes under $10,000. This was the highest OR among all the other demographics included in the study. Educational attainment followed a similar trend as income levels. Participants with a high school degree had 53% higher odds of being digitally ready (OR 1.53) than those without a high school degree, and those with a college degree or more had five times higher odds of being digitally ready (OR 5.89) compared with their less-than-high-school participants.

### 3.2. Digital readiness impact on retention of participants in the program at FQHCs

As described earlier, most of activities required by *All of Us* that qualify a participant to be considered retained (except submitting bio samples to Biobank) are completed on a web-based portal. The online portal can be accessed by the participants when they come in-person to the FQHCs or virtually from a computer or a mobile device. The instructions for participants to complete the retention activities are often sent *via* mail as paper copies, or electronically by email or text messages, based on the participant's preferences indicated when joining the Program. Therefore, digital readiness plays a key role in the ability to retain participants in the Program.

**Figure 3:**
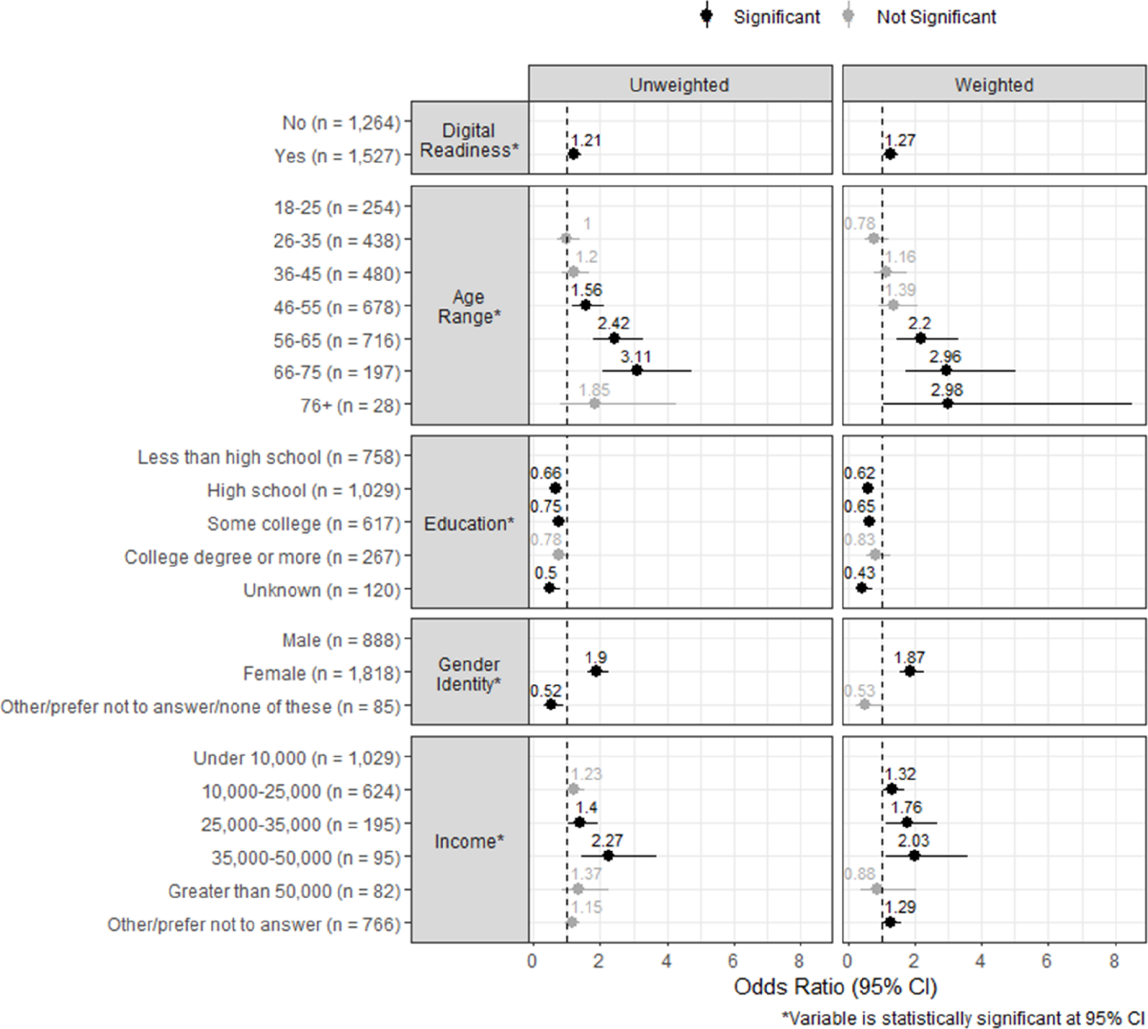
Results from weighted and unweighted models to understand the impact of digital readiness on retaining participants at FQHCs. (Outcome variable: Retention Status. Coded as 1 if the participant was retained, and 0 if the participant was never retained. Retained participants complete follow-up surveys on a web-based portal at least once every 18 months after their enrollment, indicating sustained engagement after recruitment to the Program).

Results showing the retention impact of digital readiness on FQHC participants in *All of Us* are shown in Figure 3 (Model 2). Significant variables from Model 1, which included age when consenting to the Program, income, educational attainment, and gender identity, were used for propensity score weighting in Model 2. Results from both unweighted and weighted (using IPTW propensity score methodology) models are shown. A complete table containing OR and 95% CI values for both unweighted and weighted models is included in the Supplementary Table S2 of this paper.

Digital readiness significantly increased the odds of a participant being retained in the Program; the odds were *21% higher* with the unweighted model (OR 1.21) and *27% higher* in the case of IPTW model (OR 1.27). Additionally, the odds of never being retained were significantly associated with lower age at consent, lower income, and participants identifying as male.

The odds of being retained in the Program overlapped with participant groups with higher digital readiness (i.e., higher income and participants identifying as female), except for age at consent and educational attainment. Older participants had higher odds of being retained, even though they were less digitally ready than their younger peers. Participants who were 56–65 years of age at consent had *two times higher* odds of being retained (OR 2.2) than those who were 18–25 years of age at consent; at 66–75 years of age at consent, they had *three times higher* odds (OR 2.96). This observation suggests that older participants often completed retention activities through in-person appointments with FQHC staff during their clinical visits. Older participants might be making more frequent in-person clinical visits to the FQHCs and/or may have more time available, thereby providing more opportunities for the *All of Us* staff to engage them in-person for completing retention activities. Increase in education level did not increase the odds of being retained.

Findings from Models 1 and 2 indicate that participants who were digitally ready had 27% higher odds of being retained in the Program than those who were not digitally ready. Participants with higher income, higher educational attainment, and from lower age groups were all associated with digital readiness. While the odds of digital readiness increased with increase in education level, it did not increase the odds of being retained. Participants who were not digitally ready, such as those from older age groups, but made in-person clinical visits to the FQHCs benefited from *All of Us* staff supporting them in completing retention activities, thereby compensating for their lack of digital readiness. The models also suggest that younger participants, despite their association with being digitally ready, had lower odds of ever being retained, indicating that motivating participants from younger age groups to complete the Program retention activities at FQHCs is a significant challenge, particularly as they may be making less frequent visits for healthcare.

### 3.3. Qualitative findings on the strategies FQHCs are using for retaining participants in *All of Us*

In June 2022, 11 FQHC *All of Us* staff members participated in a focus group to share their strategies for retaining participants in the Program. The strategies were focused on pre-pandemic scenarios given the scope of the analysis included in this paper, but some of the findings could be applicable during the pandemic. The key takeaways are summarized in Table 5.

**Table 5:** Summary of Strategies used by FQHCs for Retaining Participants in *All of Us*.

Strategy	Description
Continuity	Continuity strategies, such as sending birthday/Program anniversary cards to participants, or having FQHC *All of Us* staff members who initially enrolled participants call the same participants, helped build and strengthen connections. This encouraged participants who were not digitally ready to complete retention activities in-person.
Align with clinical appointments	Aligning the completion of retention activities with clinical appointments enabled completion of retention activities in-person. This strategy also saved time, as the participants could complete the activities while waiting in the lobby prior to getting called in for their clinical appointment. If the participant had more activities to complete, the nurse brought the participant back to the FQHC *All of Us* staff member after the clinical appointment concluded.
Familiarity	Familiarity with the FQHC *All of Us* staff member made a significant difference in scheduling in-person appointments to complete retention activities. Participants were more open to visiting the FQHC and resulted in fewer missed appointments with All of Us staff members.

Many of the strategies described in Table 5 further strengthen the findings from quantitative analyses. FQHC focus group members shared that aligning *All of Us* activities with clinical appointments was an especially effective strategy for older participants since they typically made more frequent in-person visits to FQHCs. They added that older participants enjoyed the company of having someone to talk to, liked to stay longer, and appreciated the service and personalized attention. This point further strengthens findings from the quantitative analysis that digital accessibility disposition for older participants had low to no impact on being retained into the Program at FQHCs.

Familiarity with a participant's digital readiness was another strategy that provides additional insights on the quantitative results. FQHCs shared that the *All of Us* staff recorded detailed notes from prior appointments about whether the participant completed all retention activities independently (vs. needing staff assistance) and their comfort using technology to determine the level of assistance needed. This allowed for the staff to be well prepared to support participant needs for completing Program activities.

## 4. Conclusions

The data presented in this paper demonstrate significant overlap between participants who are not digitally ready and those with low income, who are less educated, and of increased age. The representation of these UBR groups in clinical trials, along with longitudinal data collection, is critical to designing medical countermeasures that benefit the entire United States population and can potentially provide inference for populations around the globe. Longitudinal data collection efforts can embed measures to mitigate this disproportionate impact on UBR populations. Opportunities exist in the provision of culturally sustaining outreach and engagement to support retention, mitigating lack of digital readiness by ancillary services that bridge the gap between *All of Us* and participants who are not digitally ready, or provision of internet or internet-accessing devices to vulnerable groups.

## 5. Limitations

There were some limitations to this study, primarily due to constraints on the study design. The MCM survey questionnaire was not developed specifically for this research study. It was developed to understand the general characteristics of the population groups that FQHCs enroll. Therefore, our study was limited by the data that was already collected.

The MCM survey questions were asked by FQHC staff to all participants at their time of enrollment in *All of Us*. However, per the IRB requirements, participants were given a choice to decline responding to the MCM survey entirely or skip any of the questions. This may have introduced bias in our study sample. Out of a total of 3,552 participants enrolled by FQHCs during the study time-period, 2,897 participants chose to respond to MCM survey (82% response rate). Of the 2,897 that responded, 106 participants skipped one or more of the three MCM technology questions and were excluded (3.6%), potentially introducing selection bias. For example, these participants could have skipped the questions because they may not have access to technology devices (computers, tablets, mobile phones, and other devices) and were not comfortable stating it on the survey. Had these limitations not existed, we hypothesize that the magnitude of the quantified impact would only be greater.

The three MCM questions on technology access and participants' comfort level to using technology were used to develop a definition for digital readiness. While there is no universally established definition for digital readiness, the definition used in the study deviates from previous studies, which may limit the study's comparability with others. Some studies have used the term “digital divide”, focused on the gap between those who do and do not have access to information technology, regardless of their comfort with technology (24). Another definition of digital readiness is based on technology access, comfort level and trust (25).

Finally, the results of this study must be considered in the context of this longitudinal data collection effort, and may not generalize to other research efforts, each of which has its own definition of retention, incentive structure, and may have a vastly different study population. However, despite the limitations, the study provides timely and insightful contribution by quantifying the impact of digital readiness in recruiting and sustaining UBR population groups in longitudinal data collection.

## Data Availability

The data analyzed in this study is subject to the following licenses/restrictions: The datasets generated during and/or analyzed in this study are available from the corresponding author on reasonable request, and subject to approval from NIH. Requests to access these datasets should be directed to skini@mitre.org.
